# The *OSMR* Gene Is Involved in Hirschsprung Associated Enterocolitis Susceptibility through an Altered Downstream Signaling

**DOI:** 10.3390/ijms22083831

**Published:** 2021-04-07

**Authors:** Tiziana Bachetti, Francesca Rosamilia, Martina Bartolucci, Giuseppe Santamaria, Manuela Mosconi, Serenella Sartori, Maria Rosaria De Filippo, Marco Di Duca, Valentina Obino, Stefano Avanzini, Domenico Mavilio, Simona Candiani, Andrea Petretto, Alessio Pini Prato, Isabella Ceccherini, Francesca Lantieri

**Affiliations:** 1 Laboratory of Developmental Neurobiology, Dipartimento di Scienze della Terra dell’Ambiente e della Vita (DISTAV), Università di Genova, Viale Benedetto XV, 5, 16132 Genova, Italy; tiziana.bachetti@unige.it (T.B.); valentinaobino@gmail.com (V.O.); candiani@unige.it (S.C.); 2 UOSD Laboratorio di Genetica e Genomica delle Malattie Rare, IRCCS Istituto Giannina Gaslini, Via Gerolamo Gaslini, 5, 16148 Genova, Italy; GiuseppeSantamaria@gaslini.org (G.S.); isa.c@unige.it (I.C.); 3 Health Science Department (DISSAL), Biostatistics Unit, Università di Genova, Via Pastore 1, 16132 Genova, Italy; rosamilia.francesca@libero.it; 4 Core Facilities-Clinical Proteomics and Metabolomics, IRCCS, Istituto Giannina Gaslini, Via Gerolamo Gaslini 5, 16147 Genova, Italy; martinabartolucci@gaslini.org (M.B.); andreapetretto@gaslini.org (A.P.); 5 Fetal and Perinatal Pathology Unit, IRCCS Istituto Giannina Gaslini, Genoa, Via Gerolamo Gaslini 5, 16147 Genova, Italy; ManuelaMosconi@gaslini.org; 6 COSR-Center for Omics Sciences, IRCCS Hospital San Raffaele, Dibit2-Basilica, 3A3, Via Olgettina 58, 20132 Milano, Italy; sartori.serenella@hsr.it (S.S.); defilippo.maria1983@gmail.com (M.R.D.F.); 7 Laboratorio di Fisiopatologia dell’ Uremia, IRCCS Istituto Giannina Gaslini, Via Gerolamo Gaslini 5, 16147 Genova, Italy; marco.diduca@unige.it; 8 Pediatric Surgery Unit, IRCCS Istituto Giannina Gaslini, Via Gerolamo Gaslini 5, 16147 Genova, Italy; StefanoAvanzini@gaslini.org; 9 Department of Medical Biotechnologies and Translational Medicine, University of Milan, Via Manzoni 113, 20089 Milan, Italy; domenico.mavilio@humanitas.it; 10 Laboratory of Clinical and Experimental Immunology, IRCCS Humanitas Research Hospital, Via Manzoni 56, 20089 Rozzano Milan, Italy; 11 Centro Bosio per la Patologia Digestiva Pediatrica, Ospedale Infantile AON SS Antonio e Biagio e Cesare Arrigo, 15121 Alessandria, Italy; apini@ospedale.al.it

**Keywords:** mucosal immunity, gut inflammation, proteomics, Whole-Exome Sequencing (WES), Hirschsprung Associated Enterocolitis (HAEC), Oncostatin-M receptor (*OSMR*)

## Abstract

Hirschsprung (HSCR) Associated Enterocolitis (HAEC) is a common life-threatening complication in HSCR. HAEC is suggested to be due to a loss of gut homeostasis caused by impairment of immune system, barrier defense, and microbiome, likely related to genetic causes. No gene has been claimed to contribute to HAEC occurrence, yet. Genetic investigation of HAEC by Whole-Exome Sequencing (WES) on 24 HSCR patients affected (HAEC) or not affected (HSCR-only) by enterocolitis and replication of results on a larger panel of patients allowed the identification of the HAEC susceptibility variant p.H187Q in the Oncostatin-M receptor (*OSMR*) gene (14.6% in HAEC and 5.1% in HSCR-only, *p* = 0.0024). Proteomic analysis on the lymphoblastoid cell lines from one HAEC patient homozygote for this variant and one HAEC patient not carrying the variant revealed two well distinct clusters of proteins significantly up or downregulated upon OSM stimulation. A marked enrichment in immune response pathways (*q* < 0.0001) was shown in the HAEC H187 cell line, while proteins upregulated in the HAEC Q187 lymphoblasts sustained pathways likely involved in pathogen infection and inflammation. In conclusion, *OSMR* p.H187Q is an HAEC susceptibility variant and perturbates the downstream signaling cascade necessary for the gut immune response and homeostasis maintenance.

## 1. Introduction

Hirschsprung’s disease (HSCR) is a chronic constipation characterized by congenital absence of enteric ganglia [1].

HSCR occurs as either familial or sporadic (up to 80%), shows high heritability, and often presents together with additional congenital anomalies [1,2].

The major gene involved in HSCR is the *RET* proto-oncogene [3,4], although several other genes and loci have also been described to be involved in a minority of cases, mainly syndromic [1,5,6].

Although HSCR is now resolved by surgery in the vast majority of cases, Hirschsprung Associated Enterocolitis (HAEC), the most serious complication in HSCR, is still life-threatening, and occurs in around one third of patients [7]. HAEC manifestations include abdominal distention, fever, and diarrhea, while histologically HAEC is characterized by crypt dilatation, mucin retention, enterocyte adherence of bacteria, leucocyte infiltration, and epithelial damage. HAEC was initially believed to be caused by intestinal mechanical obstruction, but its occurrence both before and after surgery seems to claim against this hypothesis.

Several evidences suggest HAEC is due to a combination of factors leading to loss of gut homeostasis sustained by the immune system, the defense barrier, and the microbiome [7].

Indeed, a thin intestinal epithelium cell layer, together with the mucus secreted by Goblet cells, must physically separate microorganisms from the host and mutually interact with the gut microbiota [8].

Altered goblet cell and mucus properties have been proposed in HSCR [9,10]. Moreover, higher susceptibility to experimental colitis and alterations in the epithelial barrier have been reported in mouse models of defective inflammasomes [11]. Finally, differences in microbiome patterns have been reported for HSCR [12], and HAEC patients [13,14].

The high susceptibility to HAEC in HSCR patients, the HAEC both before and after surgery, and the observation that most HSCR allied disorders have a genetic basis [1] suggest also a genetic predisposition. In support, several mouse models show symptoms that resemble HAEC, such as *EdnrB^NCC^*^−^*/*^−^ [7], and *Gfra1^hypo/hypo^* mice [15]. Both *GFRA*, and *EDNRB* are HSCR causative genes, along with *RET*. Notably, *RET* seems to have a role in the gut immunity and homeostasis [16,17,18].

However, except for gut or fecal microbiome studies, neither genetic screening nor gene expression studies based on omics approach have been attempted on HAEC yet.

Here we report on Whole-Exome Sequencing (WES) performed on 12 HSCR patients without enterocolitis and 12 patients with HAEC that allowed us to identify an HAEC predisposing variant located in the Oncostatin-M receptor (*OSMR*) gene, further investigated by proteomic analysis. Our results show striking evidence that, after stimulation with OSM, the cell line carrying the rarer HAEC associated G allele was perturbated in immune pathways and enriched in several pathways likely involved in pathogen infection and inflammation. These results confirm the hypothesis of the immune system impairment in the balance between mucosal immunity, defense barrier, and microbiome in HAEC susceptibility and highlight a role of the OSM/OSMR axis.

## 2. Results

### 2.1. Variants Detection by Whole-Exome Sequencing

Whole-Exome Sequencing (WES) of 12 HAEC (also referred to as cases) and 12 HSCR-only patients (also referred to as controls) has provided the call of 77,396 variants with read depth ≥ 10 and a call quality ≥ 10, 14,781 of which are only in HAEC patients, 14,316 exclusively in HSCR-only patients, and 48,299 in both.

No gene could explain all HAEC patients through single or multiple rare different variants absent in HSCR-only patients.

We have filtered the variants following two strategies, the first one based on their predicted effect on the protein and their low frequency in the general population, the second one based on their different distribution between HAEC and HSCR-only patients.

Thirty and 41 variants were identified by these two strategies, respectively, for a total of 71 variants located in 64 genes (Table S1). A ranking of relevance to HAEC was created based on: (i) putative effect on the protein and comparison between the allele frequency in cases and controls detected by Next Generation Sequencing (NGS) and those reported in the genome aggregation database (gnomAD, https://gnomad.broadinstitute.org/ accessed on 14 July 2020) (WES score), (ii) biological role of the gene, as surmised from literature data (biological score). Of note, although the biological relevance to HAEC has been assigned in cieco with respect to the WES relevance, 36.8% of variants in genes likely relevant to HAEC had a high WES score, in comparison to 15.4% of variants in genes only possibly or unlikely to be related to HAEC (Table S2).

We selected the 5 most promising variants, located in the *JAK3*, *OSMR*, *PRMT2*, *PIKFYVE*, and *NLRP14* genes, to undergo validation and replication by Sanger sequencing. We first confirmed all the genotypes. *PRMT2*, not confirmed for one HAEC heterozygote patient, was excluded from further investigations (Table 1).

Genotyping additional 23 HAEC and 42 HSCR-only patients revealed an almost identical allele frequency in HAEC and HSCR-only patients for the *JAK3*, *NLRP14*, and *PIKFYVE* variants. A higher frequency in cases than in controls was instead confirmed for the rs34675408 variant in *OSMR*. Enlarging the screening to 72 HAEC and 108 HSCR-only patients, an overall minor allele frequency (MAF) of 14.6% for HAEC and 5.1% for HSCR-only controls was detected (Table 1).

We genotyped rs34675408 in *OSMR* in both parents of 64 of the above samples (38 HAEC and 26 HSCR-only), for which DNA was available. Transmission Disequilibrium Test (TDT) supported an over transmission of the G variant allele to HAEC affected patients, which was transmitted 11 times out of 13 (*p* = 0.0126), but not to HSCR-only patients (2 alleles transmitted out of 7) (Table 2). 

### 2.2. In Silico Analysis of the Effects of rs34675408 on the OSMR Structure

**Figure 1:**
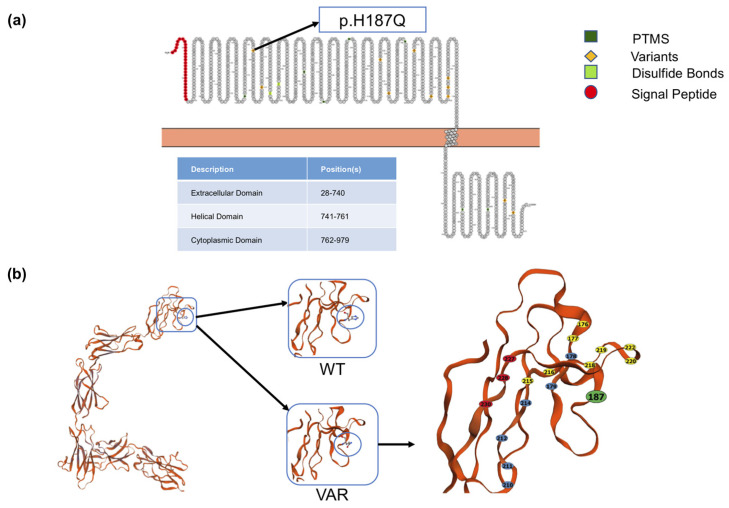
OSMR secondary and tertiary structure. (**a**) The OSMR extracellular, transmembrane, and intracellular domains are shown, as well as the protein AA sequence, as obtained with Protter. The post translational modifications (PTMs) and known variants are also detailed, including the p.H187Q SNP, indicated by an arrow; (**b**) the 3D prediction of the OSMR protein structure was obtained by the Swiss Model server using a homology method. The whole protein model is shown on the left, while the region around H187 is enlarged in the right, distinguishing between the wt (above) and the p.H187Q variant sequence (below). The region is further zoomed in to show the three hotspots sites around H187 reported by Du et al. [19] in yellow, blue, and red, respectively, with the Tyr214 residue being the hot spot binding residue in common between the three.

The OSMR protein is a component of type I and type II cytokine receptor family expressed in several tissues, including colon and rectum. The Single Nucleotide Polymorphism (SNP) rs34675408 c.561T>G is a missense variant in the exon 5 of *OSMR* leading to p.H187Q. The localization of p.H187Q in the OSM binding region of the large extracellular domain suggests that this variant could exert its susceptibility effect by modulating the OSMR activation (Figure 1).

To investigate the variant effect on the protein conformation, we compared the OSMR proteins with and without the aminoacidic change using in silico tools. The tertiary structure designed by Swiss Model [20] exhibits some divergences in the model parameters between the H187 and Q187 structures. In particular, a slightly different conformation was visible (Figure 1), with the H187 allele showing, differently from the Q187 allele, a distorted angle at the Ramachandran Plot (Figure S1). 

### 2.3. OSMR Expression in HAEC Lymphobastoid Cell Lines

To assess whether we could stimulate the OSMR mediated pathway in lymphoblastoid cells from our patients, we checked its expression by Western blot assay.

The OSMR expression was confirmed in lymphoblasts from HSCR and HAEC patients, in addition to fibroblasts, used as positive control as known to express the receptor. Moreover, the OSMR expression seemed to be even higher in the two HAEC than in the HSCR-only cell lines (Figure S2). 

### 2.4. Phospho-ERK Cellular Distribution Is Induced by OSMR Stimulation in HSCR and HAEC Lymphoblasts

The ERK phosphorylation (hereinafter pERK), downstream of the OSM-OSMR cascade, was assessed to be highly induced by OSM in the control cell line LY1765 (Figure S3).

To investigate the role of the *OSMR* SNP rs34675408 in HAEC susceptibility, we then evaluated the effect of OSM-OSMR activation in an HAEC patient homozygous for the T allele (LY3828) and in an HAEC patient homozygous for the HAEC associated G allele (LY4759) by pERK immunofluorescence analysis.

**Figure 2:**
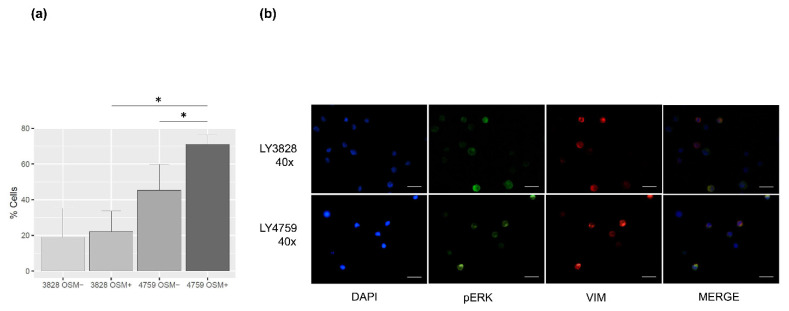
Perinuclear inclusions in H187 (LY3828) and p.H187Q homozygous variant (LY4759). (**a**) The amount of perinuclear formations are represented as means and standard deviations of the percentage of cells with inclusions obtained by three independent experimental session replicates. The inclusions percentage is higher in the p.H187Q homozygous HAEC LY4759 cell line than in the LY3828 cell line. The amount of inclusions is significantly increased by OSM treatment in LY4759 cells only. Statistical differences are indicated by an asterisk (* *p* < 0.0001); (**b**) immunofluorescence images of the OSM treated LY3828 and LY4759. Almost all the LY4759 cells showed round formations with the same localization as anti-Vim. The merge between pERK and vimentin is also displayed. The scale bar is 20 µm.

First, we observed that in many cells from both the HAEC patients pERK localized within perinuclear formations (Figure 2), which were not appreciable in the LY1765 cells. These inclusions were more frequent in the HAEC GG lymphoblasts (LY4759) than in the *OSMR* TT lymphoblasts (LY3828). This difference was more evident after OSM treatment, with “aggresome like” structures detected in 22.1% of LY3828 and 71.0% of LY4759 cells: inclusions in the latter cells significantly increased in numbers (*p* < 0.0001), while no significant change was observed in the LY3828 cells. The vimentin intermediate filaments co-localize with these pERK positive structures, but without forming the cage typically visible for classical aggresomes (Figure 2).

**Figure 3:**
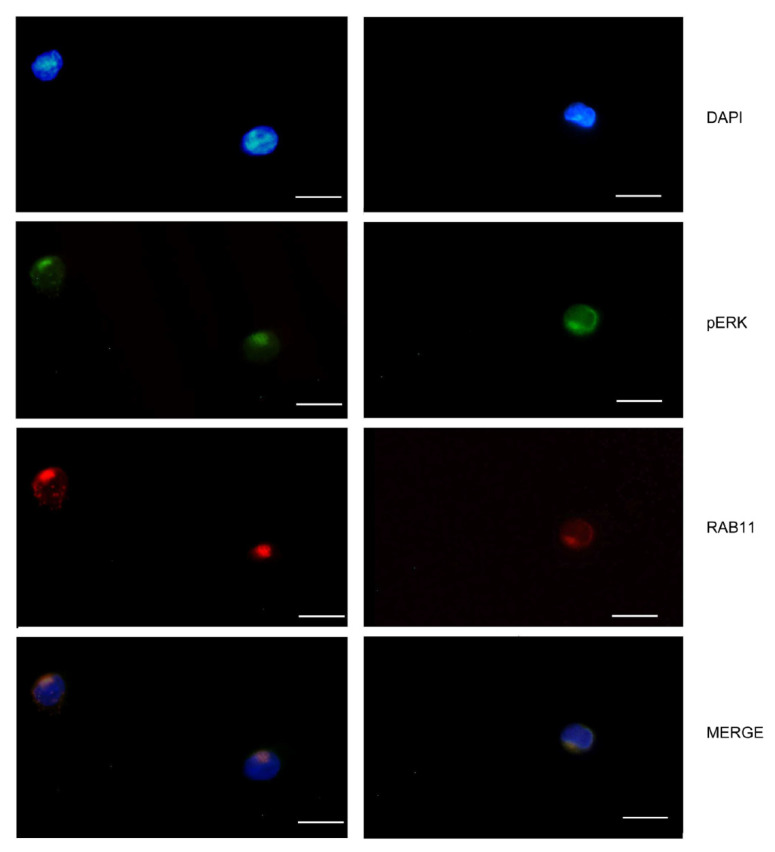
pERK localized in the same compartment with RAB11. Fluorescence images show the same cellular compartment localization of pERK with RAB11 after the OSM treatments on LY4759 cells. Two examples are reported in the two columns. The perinuclear structures in which pERK and RAB11 cluster in ERC formations are visible. The scale bar is 20 µm.

Vimentin and ERK take part in the endocytic recycling compartment (ERC), a pericentriolar membranous tubulovesicular organelle that regulates recycling to the plasma membrane. Indeed, in our cell lines Ras-Related Protein Rab-11A (RAB11), a known endocytic recycling machinery protein [21], is localized in the same compartment as pERK (Figure 3), as already described by others [22]. Our results demonstrate that the observed perinuclear structures do include ERC and that OSM treatment can enhance ERC functioning.

### 2.5. Proteomics and Pathway Analyses Reveal Pathway Perturbations Driven by the OSMR Variant

**Figure 4:**
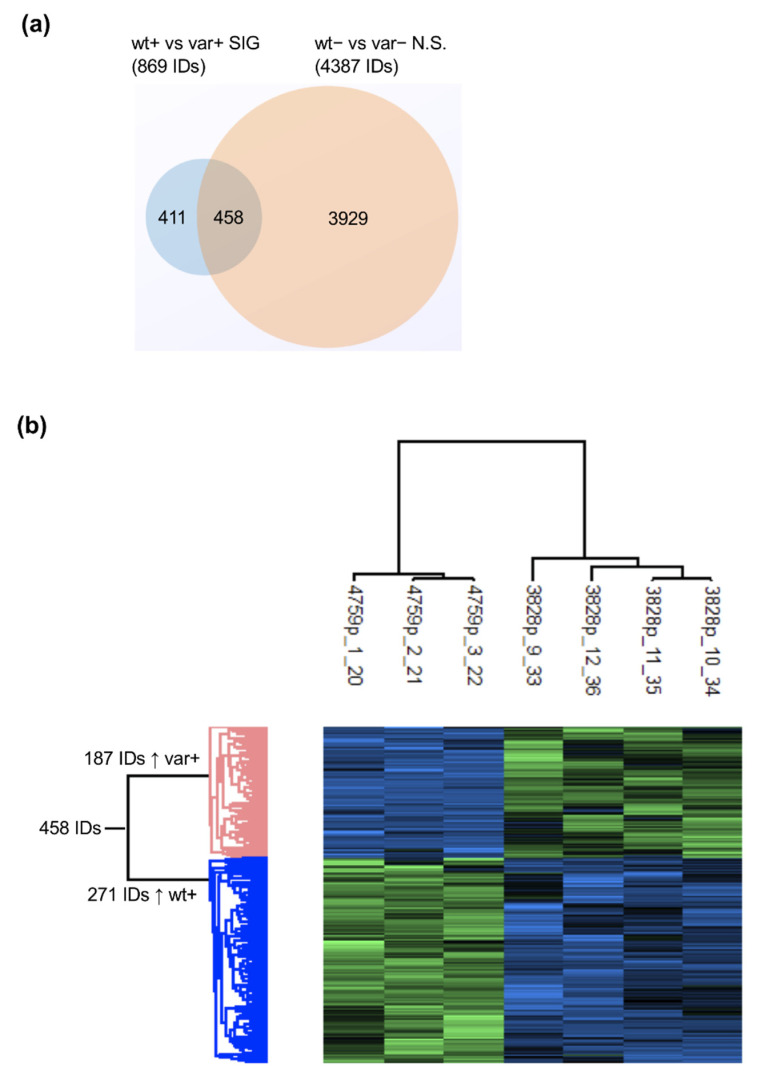
Protein cluster analysis. Cluster analysis of proteins differently expressed between the *OSMR* H187 patient’s cell line (LY3828, four replicates) and the p.H187Q homozygous patient’s cell line (LY4759, in triplicate) after OSM stimulation is shown. The two samples are referred to as wt+ and var+, respectively. (**a**) The Venn diagram represents the 458 proteins differently expressed between the wt and the var cell lines after OSM treatment (indicated by the “plus sign”) that were not significantly different between wt and var before treatment (“minus sign”); (**b**) the hierarchical analysis showed two clusters clearly visible in the heatmap for these 458 proteins: 187 proteins were overexpressed in var+ compared to wt+ (in blu in the heatmap color-scale), while 271 proteins were overexpressed in wt+ compared to var+ (in green).

To compare the OSM-OSMR cascade in the two cell lines, TT and GG, for rs34675408 we have performed a proteome analysis (Figure S4). We have detected 458 proteins that, after OSM treatment, showed significantly different intensity between the T and the G allele-carrying lymphocytes (wt+ vs. var+) by mass spectrometry. Hierarchical clustering analysis and visual heat map showed two evident clusters of proteins: 271 proteins more expressed in the wt+ than in the var+ sample, and 187 proteins more expressed in the var+ than in the wt+ sample (Figure 4).

**Figure 5:**
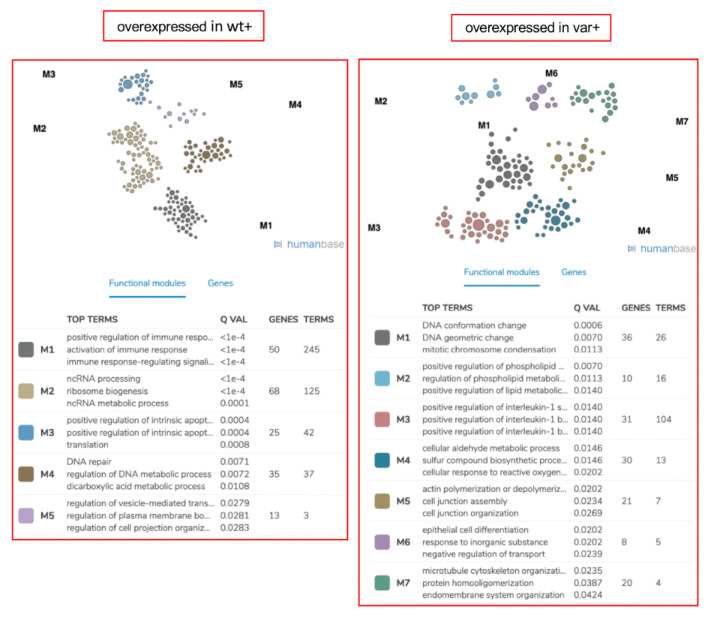
Pathway analysis in H187 (LY3828) and p.H187Q homozygous variant (LY4759) cells. The screenshot of the pathway analysis performed with HumanBase on the clusters of proteins obtained by Nano-LC mass spectrometry is shown. The 5 modules of pathways enriched based on the cluster of protein overexpressed in wt+ with respect to var+ are on the left, while the 7 enriched pathway modules in the cluster of protein overexpressed in var+ with respect to wt+ are on the right.

At the pathway analysis, the proteins overrepresented in the wt+ set showed a significant enrichment of immune response pathways, completely lost in the cluster of proteins overrepresented in var+ (Figure 5). In particular, the module M1 (*q* < 10^−4^) included 245 terms, several of which related to immune response, antigen receptor-mediated signaling, T cell activation and regulation, toll-like receptor 3 (TLR3) signaling, viral processes and defense response to organisms and peptides, response to interferon-alpha and -gamma, ERK1/ERK2 cascades, autophagy, and response to wounding, although the latter three pathways were borderline with significance.

The second most significant module (M2) regarded RNA and cell mitosis, while the M3 module mainly included proteins involved in the regulation of apoptosis, the response to DNA damage stimulus, and catabolic processes. Finally, pathways related to DNA repair and replication, protein complex disassembly, and cell polarity (M4), in addition to pathways regarding the regulation of vesicle-mediated transport and of cell projection organization (M5) were enriched in the wt+ vs. var+ proteins cluster.

In the set of protein overexpressed in the var+ sample, at opposite, there was significant enrichment in pathways related to DNA conformation, also present in the wt+ cluster but at a less extent (M1), to lipid metabolism and kinase activity (M2), and to interleukin-1 production and regulation, tumor necrosis factor-mediated signaling pathway (TNF), -Jun N-terminal Kinase (JNK) cascade, and positive regulation of stress-activated Mitogen-Activated Protein Kinase (MAPK) cascade (M3). Other enriched pathways were related to response to oxidative stress and sulfur compound biosynthetic and metabolic process (M4), actin and cell junction organization (M5), negative regulation of transport, epithelium differentiation and development (M6), and microtubule cytoskeleton organization (M7).

In an attempt to specifically select those proteins not affected by OSM activation in the presence of the *OSMR* SNP variant (hereinafter lost-effect proteins), we further filtered from the wt+ overexpressed and the var+ overexpressed clusters those proteins not differently expressed between var- and var+. The wt+ overrepresented cluster protein gave results similar to those obtained with the larger panel (243 out of 271 proteins), but a new enriched pathway regarding the unsaturated fatty acid biosynthetic process emerged (Figure S5). The var+ overrepresented cluster, although representing only a slightly smaller group (184 out of 187 proteins), showed instead additional significantly enriched pathways related to the glutathione metabolic process, detoxification, endoplasmatic reticulum (ER), and in particular ER stress, transport, and retrograde transport and degradation.

## 3. Discussion

Enterocolitis can occur both before and after a resolving surgery, thus suggesting that genetic predisposing factors play a role in the pathogenesis of the disease. However, only a few studies have searched for a genetic basis of HAEC so far, mainly through characterization of mouse models [7,15].

In this work, we report on a Whole-Exome Sequencing (WES) performed on 12 HAEC patients and 12 HSCR patients without enterocolitis that allowed us to identify *OSMR* as a HAEC candidate gene, further investigated by proteomics. 

Both the WES and the proteomic analyses are applied here for the first time to investigate the HAEC genetic susceptibility.

The *OSMR* gene is expressed in many tissues and plays an important role in various biologic functions, including cell growth, neuronal development, and inflammatory responses. *OSMR* has also been involved in several inflammation related diseases and cancers [23,24].

In particular, growing evidences show a role for the OSM-OSMR pathway in gut inflammation [25,26]. OSM and OSMR expression are higher in inflammatory bowel diseases (IBDs) patients than in controls and in the inflamed than non-inflamed lesions of IBDs patients [27,28]. IBDs share similar clinical manifestations and abnormal intestinal mucosal barrier function with HAEC and the increased susceptibility to develop IBDs in HSCR patients suggest that common mechanisms may underlie their pathogenesis [9].

The H187 residue is located in the region linking three hot spot sites of the OSM-OSMR binding task [19]. The change from basic histidine to polar glutamine, although not directly affecting a binding hot spot, could alter the conformation of the sites active in OSM-OSMR interaction. The HAEC associated G allele seems to abrogate the distorted angle in the protein conformation created by the T allele. This might amplify the OSM effect, as suggested in type 2 inflammation for gain of function SNPs in OSM and OSMR [29].

Both case-control and family-based association analyses support the OSMR SNP rs34675408 as an HAEC susceptibility variant. Unfortunately the relatively low frequency of the SNP, the HSCR low incidence and the difficulties in collecting a proper HAEC sample makes it uneasy to investigate this complication from a genetic point of view. Moreover, we cannot exclude that this variant is a confounding factor rather than predisposing to HAEC.

However, the proteomic analysis to compare the consequences of OSM treatment on the two alleles was performed on two HAEC patients, one homozygote for the *OSMR* G allele and the other a T homozygous HAEC patient. Since two clearly distinct clusters of proteins emerged after OSM induction, this variant seem to be per se involved in biological pathways likely related to HAEC occurrence.

The most significantly enriched pathways in the cluster of proteins more expressed in HAEC TT than in HAEC GG cells were related to the immune system response. This result is in accordance with a previous observation in intestinal epithelium cells [27]. Enriched pathways included positive regulation of TLRs signaling, TNF production, regulation and response, as well as Nuclear Factor Kappa-Light-Chain-Enhancer of Activated B cells (NF-kB) positive regulation. Additionally, several pathways involved in the regulation of the viral process, defense response, and interaction with host were enriched in the wt+ TT cell line.

In HAEC GG cells, no immune response pathway was enriched. At opposite, negative regulation of NF-kB emerged. Interestingly, NF-kB has proinflammatory but also tissue-protective functions in the intestinal epithelium [30]. In agreement with the inflammation cascade activated in response to pathogen attack, the positive regulation of MAPK, JNK and Interleukin 1 beta (IL-1β) was enriched in the *OSMR* SNP variant homozygous patient, likely due to the overexpression of the Apoptosis-associated speck-like protein containing a CARD (ASC) proteins PYCARD and CARD8. 

Of note, among the “lost-effect” proteins, we also observed that autophagy and fatty acid process pathways, enriched among the TT cells, were down-regulated in HAEC GG cells, while sulfur biosynthesis was more represented in GG lymphocytes. Autophagy plays crucial functions in inflammation and regulates inflammasome-mediated IL-1β production [31]. Similarly to what has been observed in TNFalpha-Receptor Associated Periodic Syndrome (TRAPS), such excessive IL-1β production could link autophagy impairment to the anti-TNFalpha therapy inefficacy [32]. OSM and OSMR have been proposed as a therapeutic target for IBDs, since their overexpression seems to be associated with ineffective anti-TNF therapy [28].

Sulfur has important physiological functions, but hydrogen sulfide is toxic and might impair colonocyte butyrate oxidation. Short-chain fatty acids (SCFAs) (e.g., butyrate) increase mucin expression, and protect the intestinal barrier [33]. Both impaired SCFA production and H_2_S are implicated in colon inflammation. A gut altered fecal SCFAs profile has been reported in HAEC [34], while goblet cell altered functions, described in HSCR [10], seem to precede the immune involvement in HAEC mice model [15].

Finally, a wound healing pathway resulted to be enriched in *OSMR* wt lymphoblasts, but not in GG lymphoblasts. Intestinal epithelial lesions are key features of IBD and also suggested in HAEC susceptibility. Following damage, the intestinal epithelium undergoes a wound healing process, possibly through Signal transducer and activator of transcription 3 (STAT3) [35], CAV1-mediated Ca(2+) signaling [36], or OSM and SERPINs [27]. Caveolin-1 (CAV1), involved in mediating bacterial and viral immunity, and in ERK1/2, and TLR4 negative regulation [37], are less expressed in colon of HSCR patients compared to controls [38] and are more expressed in TT than in GG cells. Accordingly, JNK and MAPK positive regulation pathways were enriched only in the TT cells. ERK1/2 mediate multiple cellular processes, including inflammation [39], through Ras/Raf/MAPK signaling and JNK activation. In our cellular model, following OSM treatment, pERK showed a juxtanuclear localization in many *OSMR* GG lymphocytes, while it presented the canonical diffuse cytoplasmic localization in the vast majority of TT cells. ERK localized in the same compartment with vimentin and the small GTPase Rab11, a marker of the endocytic recycling compartment (ERC). ERC is associated with the microtubule organizing center. In accordance, pathways involving actin and microtubule organization have been found enriched in GG cells. Given that ERC is involved, together with vimentin, in the recycling of membrane proteins such as integrins, ERK perinuclear localization might result in the alteration of the integrins pathway, and therefore, in the cell motility modification, thus affecting inflammation and infections. In this light, a dysregulated endocytosis pathway in HAEC *OSMR* variant cells, suggested by down-regulation of CAV1, does imply consequences both in recycling and in immune response.

## 4. Materials and Methods

### 4.1. Patients

We retrospectively checked for enterocolitis occurrence in all the consecutive patients admitted at the Gaslini Institute, Genova, Italy, with HSCR confirmed by biopsy from 1989 to 2014. HAEC diagnosis and severity were in accordance with the Pastor [40] and Elhalaby [41] criteria. This study is in accordance with the Helsinki Declaration of 1964 and later versions and was approved by the Institutional Ethical committee.

For a portion of these patients a complete phenotypic screening had been performed in the ambit of a prospective observational study [2].

We selected the HAEC patients to get sequenced by WES based on (i) presence of enterocolitis, (ii) complete phenotypic screening resulted in the absence of additional anomalies; (iii) Italian ancestry (both parents Italian); (iv) sufficient and not degraded DNA. Excluding patients for whom surgical complications and multiple surgeries might have increased HAEC risk, 12 HAEC patients were recruited.

We selected the same number of HSCR patients without enterocolitis occurrence and with similar characteristics to the HAEC group in terms of gender, length of aganglionosis and familial occurrence of HSCR, following the same criteria.

For the replicate analysis we included also patients with additional anomalies or incomplete phenotype screening, given that we were following up the HAEC complication testing only specific variants, despite a putative more complex genetic background. Up to 72 HAEC and 108 HSCR-only patients, depending on the variant, were screened (see Table S3 for patients’ characteristics).

### 4.2. Whole-Exome Sequencing and Variants Filtering, Prioritization, and Validation

Genomic DNA was extracted from lymphocytes or from immortalized lymphoblast lines by standard protocol.

Whole-Exome Sequencing (WES) was performed on the HiSeq 2500 (Illumina, San Diego, CA, USA) after enrichment library preparation with Nextera Rapid Capture Expanded Exome (Illumina). WES sequencing, the basic bioinformatics analysis and vcf file generation were endowed by the Center for Translational Genomics and BioInformatics, San Raffaele Scientific Institute.

Filtering and case-controls comparison were performed with SnpSift.

Variants with read depth ≥ 10 in all samples and a call quality ≥ 10 were filtered based on (i) low frequency (≤0.01) and the impact on the protein; and (ii) a very different distribution between cases (HAEC) and controls (HSCR-only).

To prioritize the identified variants, we assigned a scale of relevance (WES score) based on the variants annotations (impact, deleteriousness prediction, frequency in the public databases, different distribution between HAEC and HSCR-only) and a scale of biological relevance (biological score) based on the genes’ function and involvement in immunity and intestine reported in public databases and PubMed. Variants located in genes, often reported as mutated during exome analysis and thus likely to be false reports, were excluded [42].

The WES and the biological scores were multiplied to get a final ranking (Supplementary Methods and Table S1).

Selected variants validation and follow-up genotyping have been performed by Sanger sequencing.

Additional details on sequencing and variants filtering and prioritization are provided in the Supplementary Methods and Tables S1 and S4.

### 4.3. In Silico Analyses of OSMR

Protter (http://wlab.ethz.ch/protter/start/ accessed on 28 January 2020) [43] and Swiss Model, Swiss Institute of Bioinformatics, Lausanne, Switzerland (https://swissmodel.expasy.org/ accessed on 14 July 2020) [20] software were used to predict the OSMR protein secondary and tertiary structure based on the Protein Data Bank (PBD) 3l5h.1A model template and to design the Ramachandran plot, which was further investigated by the RAMPAGE software (http://mordred.bioc.cam.ac.uk/~rapper/rampage.php accessed on 14 July 2020).

### 4.4. Cell Cultures Preparation

Lymphoblastoid cell lines, produced by Epstein–Barr virus (EBV) immortalization, and fibroblast cell lines were available at the IRCCS Gaslini (Genova, Italy) from both HSCR and HAEC patients. In detail, we used lymphoblasts from a patient affected by a disorder unrelated to HSCR as control (LY1765), an HSCR-only patient (LY3956) TT homozygote for the *OSMR* wild type allele of the p.H187Q SNP, an HAEC patient (LY3828) TT homozygote for the same SNP, an HAEC patient (LY4579) GG homozygote for the variant allele of the *OSMR* p.H187Q SNP, and fibroblasts from a patient affected by a hematological disease not related to HSCR. Cell cultures were grown by standard protocols. Cells were then plated and stimulated for 30 min with 50 ng/mL Oncostatin M (OSM) (Cat.PHC5015, no. L0216061917, Invitrogen) [44] to undergo immunofluorescence assay [45] and mass spectrometry (MS) run.

### 4.5. Western Blotting Assay

Cell lines were plated in flasks and expanded to 3 × 10^6^ cells for lymphoblasts and to 1 × 10^6^ for fibroblasts. After 48 h, cells were washed with Phosphate Buffered Saline (PBS) 1×, centrifuged, and lysed with RIPA buffer (Tris–HCl 50 mM pH 7.5, NaCl 150 mM, Triton-X 1%, SDS-20 0.1%, Na deoxycholate 1%) and Protease Inhibitor. Total cell lysates were quantified with BSA assay and equal amounts were electrophoresed using Mini-PROTEAN Precast Gels and then Trans-Blot Turbo Transfer System for transferring. Proteins were identified by probing the membrane with the rabbit anti-OSMR antibody (PA5100298, Life Technologies, dilution 1:1000), specifically addressed against the OSMR protein and then with a goat anti-rabbit IgG-488 (Sigma-Aldrich, Merck, Darmstadt, Germany) dilution 1:20,000. Signals were detected using the chemiluminescence reagent ECL advance (BIORAD- Segrate (MI)-Italy) and protein levels in each sample were evaluated by comparison with corresponding amounts of the housekeeping α-tubulin with the mouse anti Tubulin antibody (Sigma-Aldrich #T5168, dilution 1:2500).

### 4.6. Immunofluorescence

Immunoassays were performed by incubating with mouse anti-MAP kinase activated (Diphosphorylated ERK -1&2, cat. M9692, no. L089M4838V, Sigma-Aldrich), rabbit anti-vimentin (cat. HPA001762. no. LB114381, Sigma-Aldrich), or rabbit anti RAB11FIP5 primary antibodies (cat. HPA036407, no. LA104076, Sigma-Aldrich). Cells were then washed with PBS and incubated with Goat anti-Mouse IgG (H+L) Highly Cross-Adsorbed Secondary Antibody, Alexa Fluor −488 (cat. A11001, no. L12599373, Invitrogen), and Goat anti-Rabbit IgG (H+L) Highly Cross-Adsorbed Secondary Antibody, Alexa Fluor 555 (cat. A21429, Invitrogen). Finally, cells were examined with Olympus IX70 microscope by using a 40× oil objective lens; images were acquired by the ColorViewII digital camera and analyzed with the Olympus Soft Imaging Systems GmbH software, Münster, Germany.

Cell culture and immunofluorescence assays followed standard protocols. See Supplementary Methods for details and for antibody incubation description.

### 4.7. Sample Preparation, NanoLC, and Mass Spectrometer Setup

MS run and analysis were performed by the Core Facility unit of the Giannina Gaslini Institute on the HAEC GG (LY4579) (var) and the HAEC TT (LY3828) (wt) patients, four replicates for both the “untreated” (wt− and var−) and the “OSM treated” (wt+ and var+) conditions (16 runs in total).

Sample preparation [46], NanoLC, and MS setup are described in Supplementary Methods. The intensity values were extracted and statistically evaluated using the ProteinGroup Table and Perseus software, Max Planck Institute of Biochemistry, Martinsried, Germany, version 1.6.10.50 [47]: the flowchart analysis is represented in Figure S4, and detailed in Supplementary Methods. Outliers were checked by Principal Component Analysis (PCA) (Figure S6) and excluded by the following analyses. We compared only proteins detected in at least three replicates in one or more of the four groups: wt−, wt+, var−, and var+. To identify proteins differently expressed after OSM activation, proteins significantly differently expressed in wt+ vs. mut+ (*q* < 0.05) were filtered, excluding those already significantly different before treatment with OSM (wt− vs. mut− n.s.). In an attempt to specifically select those proteins for which OSM activation leads to a loss of effect in the presence of the *OSMR* SNP variant, we further filtered proteins not significantly different between var- and var+ (Figure S4).

Hierarchical cluster analysis was performed to identify clusters of proteins overrepresented in wt+ with respect to var+ and vice versa. We run the hybrid hierarchical k-means clustering algorithm provided by Perseus on the z-scores normalized data applying the software default settings, which are the Euclidean distance method and average linkage agglomeration, pre-processing with k-means, number of clusters: 300; maximal number of iterations: 10. The clusters were visualized by a color scaling heatmap.

Pathway analysis on the distinct clusters have been followed using HumanBase, Flatiron Institute, Simons Foundation, New York, NY, USA (https://hb.flatironinstitute.org/ accessed on 14 July 2020) [48], which reports the one-sided Fisher’s exact tests with Benjamini–Hochberg corrections for multiple tests’ *q*-value, searching the lymphocyte network. The Venn diagram was drawn by an online software (https://www.meta-chart.com/venn#/display accessed on 3 August 2020).

### 4.8. Statistical Analysis 

#### 4.8.1. Genetic Analysis

Variants detected by WES were compared between HAEC (cases) and HSCR-only (controls) patients by the Fisher exact test for the allelic association and by the Cochran-Armitage trend test implemented in SnipSift (one tail test, no multiple test correction); the lowest *p*-value was considered. Frequencies of variants in cases and controls were compared to those reported on gnomAD v2.1.1 for the not Finnish European controls by the proportion test, also applying Bonferroni correction for the 77,396 variants detected by WES (that is *p* ≤ 6.5 × 10^−7^).

The replicate genetic association was carried out using the Fisher exact test, two tails.

Family-based association on available trios was performed by the Transmission Disequilibrium Test implemented in PLINK 1.9 (https://zzz.bwh.harvard.edu/plink/, accessed on 11 March 2021) [49].

#### 4.8.2. ERK Activation

The number of ERK fluorescent cells containing the ERC aggregates were counted, reported as percentage with respect to all the ERK fluorescent cells, and compared between groups (treatment and cell line) by the Fisher exact test. Graphs were drawn with RStudio Software, Boston, MA, USA, Version 1.2.5033.

#### 4.8.3. Mass Spectrometry

Proteins intensities obtained by spectrometry have been compared by the two tailed *t* test implemented in Perseus, applying the Benjamini–Hochberg False Discovery Rate (FDR) correction for multiple testing (*q*-value).

Significance threshold was set at alpha = 0.05.

## 5. Conclusions

In conclusion, our results suggest that the OSM-OSMR axis, already affected in several intestinal bowel pathological conditions, is involved also in Hirschsprung Associated Enterocolitis by modulating/activating pathways with a key role in HAEC pathogenesis.

Nevertheless, association replicate of the *OSMR* variant identified here would be warranted in other HAEC cohorts to better clarify the role of the OSM/OSMR axis and the p.H185Q variant in HAEC, as well as to investigate any confounding effect and differential role in HAEC and in IBDs. Replicating proteomics and assessing the protein and mRNA expression levels of specific genes found over- or under-expressed in the present study in other cell models, including transfected cell lines and intestinal biopsies, might be the next steps in elucidating the OSMR involvement in HAEC. Since *OSMR* is a drug target gene, new interesting perspectives open up.

## Figures and Tables

**Table 1:** Variants that ranked at the top after Next Generation Sequencing (NGS) filtering and prioritization.

							NGS	Replicate		Total				
Rank	Chromosomal Position	Gene	AA	rs#	REF/ALT	gnomAD °	MAF	MAF		Genotypes (omo/HT/wt)		MAF		*p* Value *
							**Cases ^^^**	**Cases**	**Controls**	**Cases**	**Controls**	**Cases**	**Controls**	
1	chr19:17940842	*JAK3*	p.Ala1094Ala	rs3212780	G/A	0.281	0.375	0.250	0.321	3/14/17	3/21/30	0.294	0.250	0.6002
2	chr21:48069682	*PRMT2*	p.Arg229Trp	rs76937225 ^§^	C/T	0.039	0.292	-	-	-	-	-	-	-
3–4	chr5:38884071	*OSMR*	p.His187Gln	rs34675408	T/G	0.070	0.292	0.117	0.057	2/17/53	0/11/97	0.146	0.051	*0.0024*
3–4	chr2:209190632	*PIKFYVE*	p.Ser1033Ala	rs999890	T/G	0.140	0.292	0.150	0.143	2/9/21	0/6/27	0.203	0.091	0.0850
5–6	chr11:7059981	*NLRP14*	p.Arg55Gln	rs61063081	G/A	0.207	0.333	0.206	0.268	1/13/15	5/12/36	0.259	0.208	0.5581
5–6	chr11:7091569	*NLRP14*	p.Leu1010Phe	rs17280682 ^§^	C/T	0.207	0.333	-	-	-	-	-	-	-

° Non Finnish European controls; ^ Minor allele frequency (MAF) in controls was 0 for all the selected variants; * Fisher’s exact test *p*-values on NGS + replicate data; significant *p*-values (*p* < 0.05) are in italics; ^§^ rs76937225 (PRMT2) was not confirmed in one patients, rs17280682 (NLRP14) was not validated because in complete linkage disequilibrium (LD) with the selected variant rs61063081.

**Table 2:** Family-based association performed on the Oncostatin-M receptor (*OSMR*) Single Nucleotide Polymorphism (SNP) rs34675408 using the Transmission Disequilibrium Test (TDT) on Hirschsprung (HSCR) Associated Enterocolitis (HAEC) and HSCR-only available trios.

**CASES (HAEC)**							
**Gene**	**SNP**	**REF**	**ALT**	T:U *	**OR (95%CI)**	**Chi-Square**	***p*-Value**
*OSMR*	rs34675408	T	G	11:2	5.5 (1.2–24.8)	6.231	0.0126
**CTRL (HSCR only)**							
*OSMR*	rs34675408	T	G	2:5	0.4 (0.1–2.1)	1.286	0.257

* Transmitted (T) and Untransmitted (U) ratio.

## Data Availability

Data are available upon request.

## Supplementary Materials

The following are available online at https://www.mdpi.com/article/10.3390/ijms22083831/s1.

## Abbreviations

HSCR, Hirschsprung disease; HAEC, HSCR Associated Enterocolitis; WES, Whole-Exome Sequencing; OSM, Oncostatin-M; OSMR, Oncostatin-M receptor; RET, Ret Proto-Oncogene; EDNRB, Endothelin Receptor Type B; GFRa1, Glial cell line-derived neurotrophic factor (GDNF) Family Receptor Alpha-1; GnomAD: Genome Aggregation Database; NGS, Next Generation Sequencing; JAK, Janus Kinase; PRMT2, Protein Arginine Methyltransferase 2; PIKFYVE, Phosphoinositide Kinase, FYVE-type Zinc Finger; NLRP14, NLR family Pyrin Domain Containing 14; MAF, Minor Allele Frequency; Transmission Disequilibrium Test (TDT); SNP, Single Nucleotide Polymorphism; ERK, Extracellular Signal-Regulated Kinases; pERK, Phosphorylated Extracellular Signal-Regulated Kinases; ERC, Endocityc Recycling Compartment; RAB11, Ras-Related Protein Rab-11A; TNF, Tumor Necrosis Factor; JNKs, c-Jun N-terminal Kinases; MAPK, Mitogen-Activated Protein Kinase; ER, Endoplasmic Reticulum; IBDs, Inflammatory Bowel Diseases; TLRs, Toll Like Receptors; NF-kB, Nuclear Factor Kappa-Light-Chain-Enhancer of Activated B cells; IL-1β, Interleukin 1 beta; ASC/PYCARD, Apoptosis-Associated Speck-Like Protein Containing a CARD; CARD8, Caspase Recruitment Domain-Containing Protein 8; TNFalpha-Receptor, Tumor Necrosis Factor Alpha-Receptor; SCFA, Short-Chain Fatty Acids; H_2_S, Hydrogen Sulfide; CAV1, Caveolin-1; PBD, Protein Data Bank; EBV, Epstein–Barr Virus; PBS, Phosphate Buffered Saline; RAB11FIP5, RAB11 Family Interacting Protein 5; DAPI, 4′,6-diamidino-2-phenylindole; FDR, False Discovery Rate; STAT, Signal Transduction and Activator of Transcription.
