# Supplementary material for: The OSMR Gene Is Involved in Hirschsprung Associated Enterocolitis Susceptibility through an Altered Downstream Signaling
Source: Int J Mol Sci. 2021 Apr 7;22(8):3831. doi: 10.3390/ijms22083831 (PMC8067804; doi:10.3390/ijms22083831)
Supplement: Supplementary file 1 [file ijms-22-03831-s001.zip › SupplementaryMethods_IJMS_Lantieri.docx]

**SUPPLEMENTARY METHODS**

**Whole Exome Sequencing**

Exome enrichment library preparation was performed with Nextera Rapid Capture Expanded Exome version of the kit (Illumina, San Diego, CA, USA), following manufacture instruction and using 100ng of high-quality gDNA. Sequencing was performed on HiSeq2500 (Illumina), based on SBS chemistry. We generate PE (pair end) reads, 100 nt long each, to obtain in average a 50x coverage.

Sequence alignment was performed to the GRC37/hg19 reference using Burrows-Wheeler Aligner (BWA, v0.7.12). Local realignment and base quality recalibration was performed using the Genome analysis Toolkit (GATK v3.5). Variant detection was performed using FreeBayes v1.0.2 with at least 20% of observations in the alternate allele supported by at least 2 counts and a minimum base quality of 20. SNPEff (v4.1) was used for the annotation. Coverage and capture statistics were performed using Picard (http://broadinstitute.github.io/picard/).

Variant annotation was based on NSFP database.

**WES variants filtering and prioritization**

Variants with read depth ≥ 10 in all samples and a call quality ≥ 10 were filtered by two strategies:

i) “novel” variants (allele frequency ≤0.01 or ≥0.99 in the general population from public databases) with high impact on the protein (novel_high group), and “novel” variants with high or moderate impact, predicted to be deleterious and with evidence of selection constrains and conservation among vertebrates (novel_pred group). By this strategy we filtered respectively 8 and 23 variants, one of which in common to both groups, that were present in at least two cases’ alleles (HAEC patients) and absent in controls (HSCR-only).

ii) variants present in at least 6 alleles among cases and in no controls (or on 24 alleles in controls and less than 19 alleles in cases) (6_0 group) or showing p-value ≤ 0.001 in an exploratory association analysis between cases and controls (p_001 group), thus selecting respectively 30 and 12 variants, one of which in common.

In particular, we checked the minor allele frequency (MAF) against the Non-Finnish European ExAC database, or, when not available, against 1000genomes Europeans or dbSNP databases. The impact on the protein is the effect predicted by SnpEff as described by the authors, “high” for deletions, duplications or inversions, frameshift variants, stop or start codon loss or gain, “moderate” for in frame inserts or deletions and missense variants; “low” for synonymous and “modifier” for non-coding variants.

The deleterious prediction was based on Mutation Taster, Polyphen-2, and Sift, while the selective constrains and the conservation were annotated based on GERP++ RS score and phastCons100way vertebrate. To be more stringent we filtered only those variants that were predicted to be damaging or probably damaging on at least one isoform of the protein by all the three softwares and with both evidence of selective constrains and conservation among vertebrates, that is GERP++ RS ≥2 and phastCons100way_vertebrate ≥0.13.

Finally, we excluded variants located in genes likely to be erroneously reported as mutated during exome analysis [42].

To create a scale of relevance (WES score), we evaluated as more relevant the variants:

i) with high impact on the protein (except for the novel_high group);

ii) predicted as deleterious by one or more in silico methods or showing conservation or selection constrains evidences (except for the novel_pred group);

iii) present in more than 6 alleles among cases (except for the p_001 group);

iv) rare (MAF<0.05, except for the novel_high and novel_pred groups) or never reported at all in the public databases;

v) with frequencies reported in public databases similar to our HSCR-only sample but different from HAEC and in accordance with a role of the variant in predisposing to HAEC (i.e. MAF_cases_ higher than both MAF_controls_, and MAF_db_, and not in between MAF_controls_ and MAF_db_);

vi) with p-value lower than the alpha 0.001 chosen to filter variants.

MAFs of filtered variants were manually checked on the updated ExAC database, that is gnomAD v2.1.1, which distinguishes a control panel of samples.

We also searched Ensembl, genecard, OMIM, and PubMed for the biological role of the genes where the variants were localized. We considered as relevant the genes involved in inflammation, immune response, gut, and experimental colitis and IBDs. We have thus arbitrarly classified the genes as “very likely” (5 points), “likely” (4 points), “possible” (3 points), “unlikely” (2 points) and “no/unknown” (1 point) (biological score).

A final ranking obtained by multiplying the WES and the biological scores (Table S1) was thus created.

**Sequencing and genotyping**

Selected variants were validated by Sanger sequencing first in the 12 HAEC cases, and, if confirmed, in the 12 HSCR-only patients. We have replicated the association analysis for the validated variants in a larger cohort of 65 HAEC and 105 HSCR-only patients for the most promising variant in OSMR and 23 HAEC and 42 HSCR-only patients for other promising variants.

Regions spanning the selected variants were amplified by using Accuprime GC RICH kit (Invitrogen, Life Technologies) (see Table S4 for primers’ sequences). In particular, all PCR reactions specific for GC rich templates were set up in 25 μl total volume PCR reactions containing 200 ng of genomic DNA, 400 nM primers, Accuprime buffer B and run for 35 cycles with 45s 95°C denaturation, 45s 57°C annealing and 1min 72°C extension.

PCR products were checked on 2% agarose gel, purified by ExoStar and subjected to Sanger sequencing analysis using an automated ABI-3730 Sequencer (Applied Biosystems; Thermo Fisher Scientific, Inc., Waltham, MA, USA). Sequences were visualized by FinchTV.

***In silico* analyses of OSMR**

Protter (<http://wlab.ethz.ch/protter/start/>) [43] and Swiss Model (<https://swissmodel.expasy.org/>) [20] software were used to predict the OSMR protein secondary and tertiary structure and to localize the SNP in the model, also with respect to the hotspot binding sites of OSM-OSMR described by Du et al. [19].

The Protein Data Bank (PBD) 3l5h.1A model template was used as the best homolog for OSMR (corresponding to Interleukin-6 receptor subunit beta, with 21,84% identity). Three separate modelling servers further confirmed the 3l5h.1A model: Modeller (https://salilab.org/modeller/), PhyreRisk (http://phyrerisk.bc.ic.ac.uk/home) and Phyre2 (Protein Homology/analog Y Recognition Engine, <http://www.sbg.bio.ic.ac.uk/~phyre2/html/page.cgi?id=index>). In particular, Phyre2 reports that 561 residues of the query sequence (57%) were modelled with the 100% of confidence.

The 3l5h.1A model was also used to design the Ramachandran plot and the results were investigated by the RAMPAGE software for the evaluation of the plot quality (http://mordred.bioc.cam.ac.uk/~rapper/rampage.php).

**Cell cultures**

Cell cultures were grown in RPMI 1640 (Euroclone) with 10% Fetal Bovine Serum (FBS) (Sigma-Aldrich), 1% L-glutamine (Euroclone), 100 U/ml penicillin (Euroclone) and 100 g/ml streptomycin (Euroclone) at 37 °C, 5% CO2 in a humidified incubator.

Cells were plated without supplements and stimulated for 30 minutes with 50ng/ml Oncostatin M (OSM) (Cat.PHC5015, no. L0216061917, Invitrogen). The dose was chosen accordingly to literature [44], while the stimulation time was decided after performing a time-dependent immunofluorescence analysis on ERK1/2 phosphorylation using lymphoblasts from the healthy donor (LY1765). Lymphoblasts were seeded at concentration of ∼1 × 10^6^ cells/ml, pelleted by centrifugation at 250 × g for 5 min, and resuspended in PBS (Phosphate Buffered Saline Tablets, Dulbecco's Formula, 1X) for 30’ to undergo immunofluorescence [45].

**Immunofluorescence**

Cells were fixed with 4% paraformaldehyde (PFA) for 10 min at room temperature, followed by incubation with permeabilization buffer (0.1% Tryton x – 100 in PBS) for 15 min and then with blocking solution for 5’ (1% BSA, 1% TWEEN 0,1% and 5% FBS in PBS). Cells were then incubated with mouse anti-MAP kinase activated (Diphosphorylated ERK -1&2, cat. M9692, no. L089M4838V, Sigma-Aldrich), rabbit anti-vimentin (cat. HPA001762. no. LB114381, Sigma-Aldrich), or rabbit anti RAB11FIP5 primary antibodies (cat. HPA036407, no. LA104076, Sigma-Aldrich) in blocking buffer for 90 min. Cells were then washed two times with PBS and incubated with [Goat anti-Mouse IgG (H+L) Highly Cross-Adsorbed Secondary Antibody, Alexa Fluor -488](https://www.thermofisher.com/order/genome-database/details/antibody/A-11029?SID=srch-srp-A11029) (cat. A11001, no. L12599373, Invitrogen) and [Goat anti-Rabbit IgG (H+L) Highly Cross-Adsorbed Secondary Antibody, Alexa Fluor 555](https://www.thermofisher.com/order/genome-database/details/antibody/A-21429?SID=srch-srp-A21429)(cat. A21429, Invitrogen) respectively in blocking buffer or 10% FBS in PBS for 60 min.

Nuclei were stained with DAPI [1μg/μl]; slides were added with the mounting medium (ProLong™ Gold Antifade Mountant, cat. L826469, InVitrogen or Glycerol 40% in PBS), then examined with Olympus IX70 microscope by using a 40x oil objective lens.

**Sample preparation, NanoLC and mass spectrometer setup**

Mass spectrometry (MS) run and analysis were performed on a HAEC patient homozygote for the G rs34675408 variant (LY4579) and a HAEC patient homozygote for the T allele (LY3828). The two cell lines were stimulated with 50 ng/ml OSM for 30 minutes as in the immunofluorescence assay. For both the “untreated” and the “OSM treated” conditions, four samples replicates containing 1 million cells were centrifugated at 1500 rpm for 10 min. Pellets of cells were solubilized in 50 ul LYSE buffer (PreOmics) at 95°C for 10 min and sonicated with a Ultrasonic Processor UP200St (Hielscher), 3 cycles of 30 sec. Lysate samples were digested with 0.7 ug Trypsin and 0.3 ug LysC overnight at 37 °C. Digested samples were processed by iST protocol [46].

Each sample was loaded from the sample loop directly into a 200 cm uPAC C18 column (PharmaFluidics) mounted in the thermostatic column compartment and maintained at 40°C. The peptides were separated with increasing organic solvent at a flow rate of 350 nl/min using a non-linear gradient of 5-45 % solution B (80% CAN and 20% H2O, 5% DMSO, 0.1% FA) in 155 min.

**Mass spectrometry data analysis.**

Eluted peptides were analyzed using an Orbitrap Fusion Tribrid mass spectrometer (ThermoScientific). For MS1 measurements orbitrap detection was used at resolving power of 120 K, while for MS2 measurements Ion Trap detection was used with Rapid Ion Trap Scan Rate. Top speed mode with a 2 sec. cycle time was performed for data dependent MS/MS analysis, during which precursors detected within the range of m/z 375−1500 were selected for activation in order of abundance. Quadrupole isolation with a 1.6 m/z isolation window was used, and dynamic exclusion was enabled for 30s. Automatic gain control targets were 4 × 10E5 and 1 × 10E4, with 50 and 45 ms maximum injection times, for MS1 and MS2 respectively. The signal intensity threshold for MS2 was 1 × 10E4. HCD (Higher-energy Collisional Dissociation) was performed using 28% normalized collision energy. One microscan was used for both MS1 and MS2 events.

MaxQuant software [47], version 1.6.10.0, was used to process the raw data, setting a false discovery rate (FDR) of 0.01 for the identification of proteins, peptides and PSM (Peptide-Spectrum Match). For peptide identification a minimum length of 6 amino acids was required. Andromeda engine, incorporated into MaxQuant software, was used to search MS/MS spectra against Uniprot human database (release UP000005640_9606 April 2019). In the processing, the Carbamidomethyl (C) was selected as fixed modification and the variable modifications were Acetyl (Protein N-Term), Oxidation (M), Deamidation (NQ).

The intensity values were extracted and statistically evaluated using the ProteinGroup Table and Perseus software version 1.6.10.50 [47]. The algorithm MaxLFQ was chosen for the protein quantification with the activated option ‘match between runs’ to reduce the number of the missing proteins.

We have filtered out the potential contaminant, excluded proteins with too many undetected values, log2 transformed the data and imputed missing data from normal distribution (Figure S4). Two outliers, detected through the Principal Component Analysis (PCA), could be reconducted to specific factual errors in the sample handling and preparation (Figure S6). We then re-analyzed the data excluding the two outliers. By keeping only proteins with not missing values in at least 70% of replicates in each group, wt-, wt+, var-, and var+, we have actually kept proteins detected in at least three replicates in one or more groups.

The protein intensities between the two samples, wt and variant for the *OSMR* SNP, were compared by the two tails t test, applying the S=0.1 parameter suggested by Perseus for more stringent analysis and applying the Benjamini-Hochberg FDR correction for multiple testing (q-value). Proteins significantly differently expressed in wt+ vs mut+ (q<0.05) and not different before treatment with OSM (wt- vs mut- n.s.) were considered differently expressed after OSM activation. Among these, proteins not significantly different between var- and var+ at a two tail paired t-test were further filtered, although an only a little smaller group was obtained (427 out of 458 proteins) (Figure S4).

**References**

42. Fuentes Fajardo K, Adams D, Mason C, Sincan M, Tifft C, Toro C, Boerkoel C, Gahl W, Markello T (2012) Detecting false-positive signals in exome sequencing. Hum. Mutat 33: 609-613. DOI 10.1002/humu.22033

43. Omasits U, Ahrens C, Müller S, Wollscheid B (2014) Protter: interactive protein feature visualization and integration with experimental proteomic data. Bioinformatics (Oxford, England) 30: 884-886. DOI 10.1093/bioinformatics/btt607

20. Biasini M, Bienert S, Waterhouse A, Arnold K, Studer G, Schmidt T, Kiefer F, Gallo Cassarino T, Bertoni M, Bordoli L, et al. (2014) SWISS-MODEL: modelling protein tertiary and quaternary structure using evolutionary information. Nucleic acids res. 42: W252-258. DOI 10.1093/nar/gku340

19. Du Q, Qian Y, Xue W (2020) Molecular Simulation of Oncostatin M and Receptor (OSM-OSMR) Interaction as a Potential Therapeutic Target for Inflammatory Bowel Disease. Front. Mol. Biosci. 7. DOI 10.3389/fmolb.2020.00029

44. Migita K, Komori A, Torigoshi T, Maeda Y, Izumi Y, Jiuchi Y, Miyashita T, Nakamura M, Motokawa S, Ishibashi H (2011) CP690,550 inhibits oncostatin M-induced JAK/STAT signaling pathway in rheumatoid synoviocytes. Arthritis Res Ther, pp. R72.

45. Tsang M, Gantchev J, Ghazawi F, Litvinov I (2017) Protocol for adhesion and immunostaining of lymphocytes and other non-adherent cells in culture. BioTechniques 63: 230-233. DOI 10.2144/000114610

46. Kulak N, Pichler G, Paron I, Nagaraj N, Mann M (2014) Minimal, encapsulated proteomic-sample processing applied to copy-number estimation in eukaryotic cells. Nat. methods 11: 319-324. DOI 10.1038/nmeth.2834

47. Tyanova S, Temu T, Sinitcyn P, Carlson A, Hein M, Geiger T, Mann M, Cox J (2016) The Perseus computational platform for comprehensive analysis of (prote)omics data. Nature Methods 13: 731-740. DOI doi:10.1038/nmeth.3901

**SUPPLEMENTARY FIGURES LEGENDS**

**Figure S1.** Ramachandran Plot.

The Ramachandran Plot was investigated for a geometric validation of the OSMR tertiary structure model. The plot resolution correlates with the residues found inside the most favoured φ,ψ regions.

From our Ramachandran plot analysis, the residue H187 was estimated to create a bad angle (**a**), while the variant p.H187Q did not (**b**).

**Figure S2.** Western Blot Assay.

Western Blot was performed to confirm the OSMR expression in Lymphoblast cell lines from HSCR (3956) and HAEC patients (3828 and 4759). OSMR was also expressed in lymphoblasts from a patient affected by Duchenne muscular dystrophy (1765), a disease not related to HSCR but in which OSMR is known to be expressed, and from Fibroblasts (591) used as positive controls. The housekeeping expression was confirmed with a mouse anti Tubulin antibody.

**Figure S3.** Phospho-ERK fluorescence in control cells.

Phospho-ERK fluorescence in the LY1765 control lymphoblasts is shown before and after stimulation with OSM

**a** The graph representation of quantification of the pERK fluorescence cells percentage. Statistical difference between not stimulated and stimulated is shown as an asterisk (* =p<0.0001)

**b** Microscope pERK fluorescence analysis. Nuclei were stained with DAPI and images at the right end represent the merge of the two adjacent pictures. Scale bar is 20 µm.

**Figure S4.** Proteomic analysis flowchart.

Flowchart of the analysis performed with Perseus to identify the protein differently expressed between the wt TT cell line and the var GG HAEC cell lines following mass spectrometry. PCA: Principal Component Analysis.

* The replicates were supposed to be 4 each. The analysis resulted in 5 replicates for var- and 3 replicates for var+ because a label + was erroneously read as -. Although the mistake has immediately recognized after the analysis, the sample has been excluded, together with another replicate, for which we have afterward realized that an exchange between a pellet and a lysate had been made.

**Figure S5.** Pathway analysis of the OSM-lost effect proteins.

The OSM-lost effect proteins are those proteins part of the 458 differently expressed between wt and var cell lines after OSM stimulation that are specifically not differently expressed between var- and var+.

**a)** the proteins more expressed in the wt+ than in the var+ cell line and **b)** the proteins more expressed in the var+ than in the wt+ cells are shown.

The original 458 different-effect proteins analysis is represented on the right for comparison. Correspondence between the modules is indicated by the arrows, while the additional pathways that have emerged are highlighted by the surrounding rectangles.

**Figure S6.** Principal Component Analysis.

Principal Component Analysis (PCA) was performed to investigate the samples run by Mass spectrometry and check for outliers. The two samples mistaken during handling and therefore excluded from subsequent analyzes are indicated by an asterisk.
