# Supplementary material for: The OSMR Gene Is Involved in Hirschsprung Associated Enterocolitis Susceptibility through an Altered Downstream Signaling
Source: Int J Mol Sci. 2021 Apr 7;22(8):3831. doi: 10.3390/ijms22083831 (PMC8067804; doi:10.3390/ijms22083831)
Supplement: Supplementary file 1 [file ijms-22-03831-s001.zip › TableS4_IJMS_Lantieri.docx]

**Table S4.** Primers’ sequences and PCR amplimer size.

| **Primers** | **Gene** | **Rs#** | **Sequence primer 5'-3'** | **Size PCR (bp)** |
| --- | --- | --- | --- | --- |
| F | JAK | 3212780 | GCCTCAGTTTCCCAGTCTGT | 256 |
| R |  |  | CCCTCATAGGCACAGGTGTT |  |
| F | PRMT2 | 76937225 | ATCATCACCGTGTACCAGCA | 199 |
| R |  |  | CAAAATGAATCACGCACGAC |  |
| F | OSMR | 34675408 | GTGGAAGAAGGCACCAATGT | 269 |
| R |  |  | AATGAGAAGAAAGGGCCACA |  |
| F | PIKFYVE | 999890 | CTTTGCACCTGTACCGGAAT | 231 |
| R |  |  | TCCTGCTTAAAGGCCAAAGA |  |
| F | NLRP14 | 61063081 | TGGACAAGATGGCAGATTCA | 245 |
| R |  |  | CCAGGCTTTCTCTCCTGGAT |  |
