# Supplementary material for: The OSMR Gene Is Involved in Hirschsprung Associated Enterocolitis Susceptibility through an Altered Downstream Signaling
Source: Int J Mol Sci. 2021 Apr 7;22(8):3831. doi: 10.3390/ijms22083831 (PMC8067804; doi:10.3390/ijms22083831)
Supplement: Supplementary file 1 [file ijms-22-03831-s001.zip › TableS2_IJMS_Lantieri.docx]

**Table S2.** Distribution of WES scores with respect to biological relevance score assigned to the filtered variants detected by WES.

|  |  | biological relevance score | | | | |  |
| --- | --- | --- | --- | --- | --- | --- | --- |
|  |  | very likely | likely | possible | unlikely | no/unknown | tot |
| WES score | ≥ 3 (%) | 4 (44.4) | 3 (30.0) | 1 (12.5) | 3 (12.0) | 4 (21.1) | 15 |
|  |  | 7 (36.8) | | 8 (15.4) | |  |  |
|  | <3 (%) | 5 (55.6) | 7 (70.0) | 7 (87.5) | 22 (88.0) | 15 (79.9) | 56 |
|  |  | 12 (63.2) | | 44 (74.6) | |  |  |
|  | tot | 9 | 10 | 8 | 25 | 19 | 71 |

Difference between the highest and lowest scores (≥ 3 or <3), by grouping very likely and likely vs possible, unlikely and no/unknown, was borderline with significance (p=0.0548).
