# Supplementary material for: The OSMR Gene Is Involved in Hirschsprung Associated Enterocolitis Susceptibility through an Altered Downstream Signaling
Source: Int J Mol Sci. 2021 Apr 7;22(8):3831. doi: 10.3390/ijms22083831 (PMC8067804; doi:10.3390/ijms22083831)
Supplement: Supplementary file 1 [file ijms-22-03831-s001.zip › FigureS4_IJMS_Lantieri.pptx]

## Slide 1
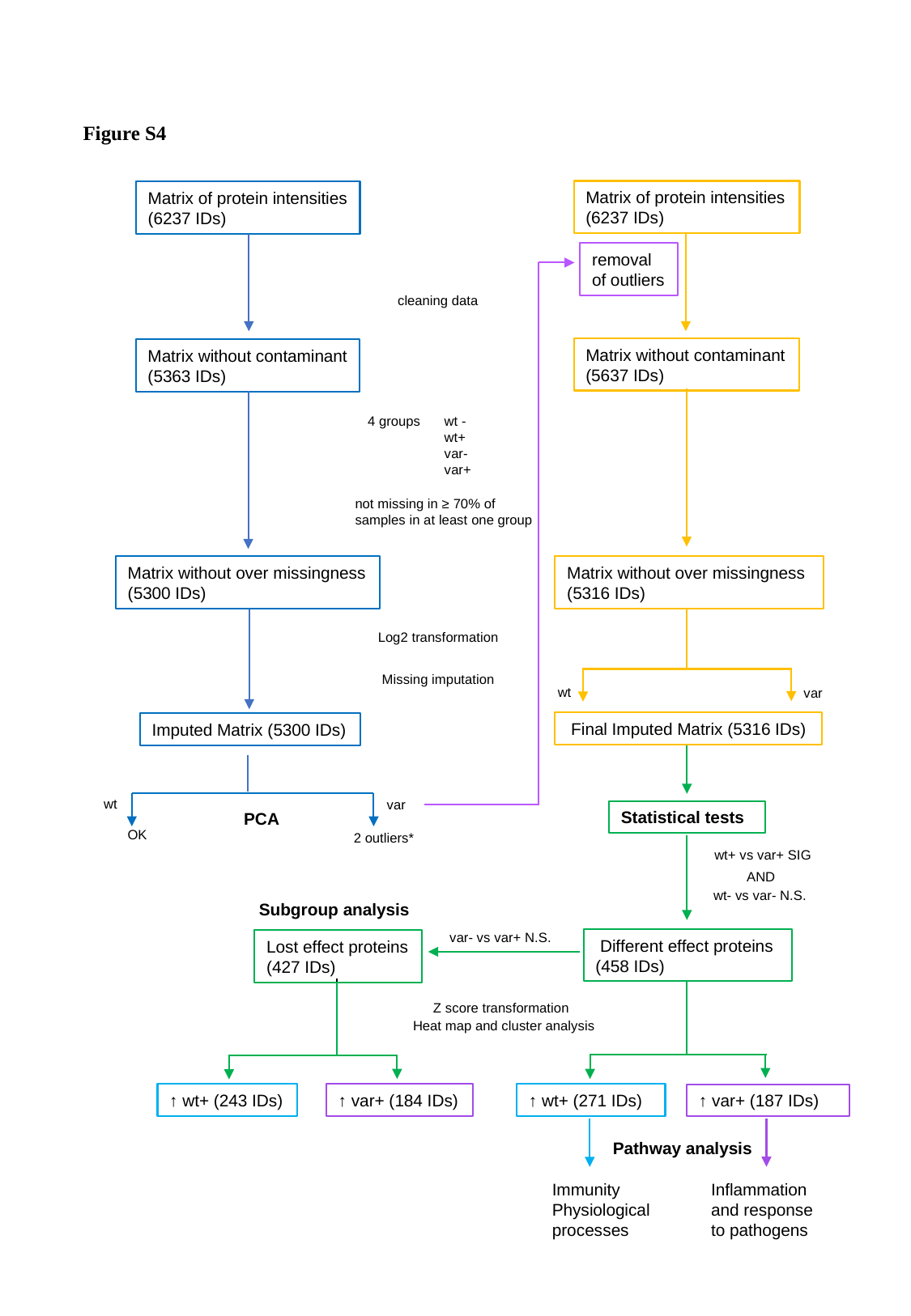

Figure S4
Matrix of protein intensities (6237 IDs)
Matrix of protein intensities (6237 IDs)
removal of outliers
cleaning data
Matrix without contaminant (5637 IDs)
Matrix without contaminant (5363 IDs)
4 groups
wt -
wt+
var-
var+
not missing in ≥ 70% of samples in at least one group
Matrix without over missingness (5300 IDs)
Matrix without over missingness (5316 IDs)
Log2 transformation
Missing imputation
wt
var
Final Imputed Matrix (5316 IDs)
Imputed Matrix (5300 IDs)
wt
var
Statistical tests
PCA
OK
2 outliers*
wt+ vs var+ SIG
AND
wt- vs var- N.S.
Subgroup analysis
var- vs var+ N.S.
 Different effect proteins (458 IDs)
Lost effect proteins (427 IDs)
Z score transformation
Heat map and cluster analysis
↑ wt+ (243 IDs)
↑ var+ (184 IDs)
↑ wt+ (271 IDs)
↑ var+ (187 IDs)
Pathway analysis
Immunity
Physiological processes
Inflammation and response to pathogens
