# Supplementary material for: The OSMR Gene Is Involved in Hirschsprung Associated Enterocolitis Susceptibility through an Altered Downstream Signaling
Source: Int J Mol Sci. 2021 Apr 7;22(8):3831. doi: 10.3390/ijms22083831 (PMC8067804; doi:10.3390/ijms22083831)
Supplement: Supplementary file 1 [file ijms-22-03831-s001.zip › TableS3_IJMS_Lantieri.docx]

**Table S3.** Clinical characteristics of patients genotyped for the rs34675408 OSMR SNP.

|  |  | HAEC (N=72) | HSCR-only (N=108) | tot* (N=180) |
| --- | --- | --- | --- | --- |
| gender M (%) |  | 59 (81.9) | 80 (74.1) | 139 (77.2) |
| HSCR-form^§^ (%) | S | 41 (56.9) | 79 (73.1) | 120 (66.7) |
|  | L | 10 (13.9) | 15 (13.9) | 25 (13.9) |
|  | TCA/TIA | 20 (27.8) | 12 (11.1) | 32 (17.8) |
|  | unknown | 1 (1.4) | 2 (1.9) | 3 (1.7) |
| familial cases (%) |  | 6 (8.3) | 7 (6.5) | 13 (7.2) |
| allied phenotypes (%) | yes | 23 (31.9) | 34 (31.5) | 57 (31.7) |
|  | no | 13 (18.1) | 25 (23.1) | 38 (21.1) |
|  | not known | 36 (50.0) | 49 (45.4) | 85 (47.2) |

* Includes both the 24 samples analyzed by WES and ascertained to be isolated HSCR, and the 156 samples analyzed at follow-up for the OSMR selected variant only.

§ The variable aganglionosis length correlates with disease severity and is classifies as Short-form (under the sigmoid), L-form (above the sigmoid), and Total Colonic or Total Intestinal Aganglionosis (TCA and TIA respectively).
