# Supplementary figures and images for: The OSMR Gene Is Involved in Hirschsprung Associated Enterocolitis Susceptibility through an Altered Downstream Signaling
Source: Int J Mol Sci. 2021 Apr 7;22(8):3831. doi: 10.3390/ijms22083831 (PMC8067804; doi:10.3390/ijms22083831)

## Slide 1
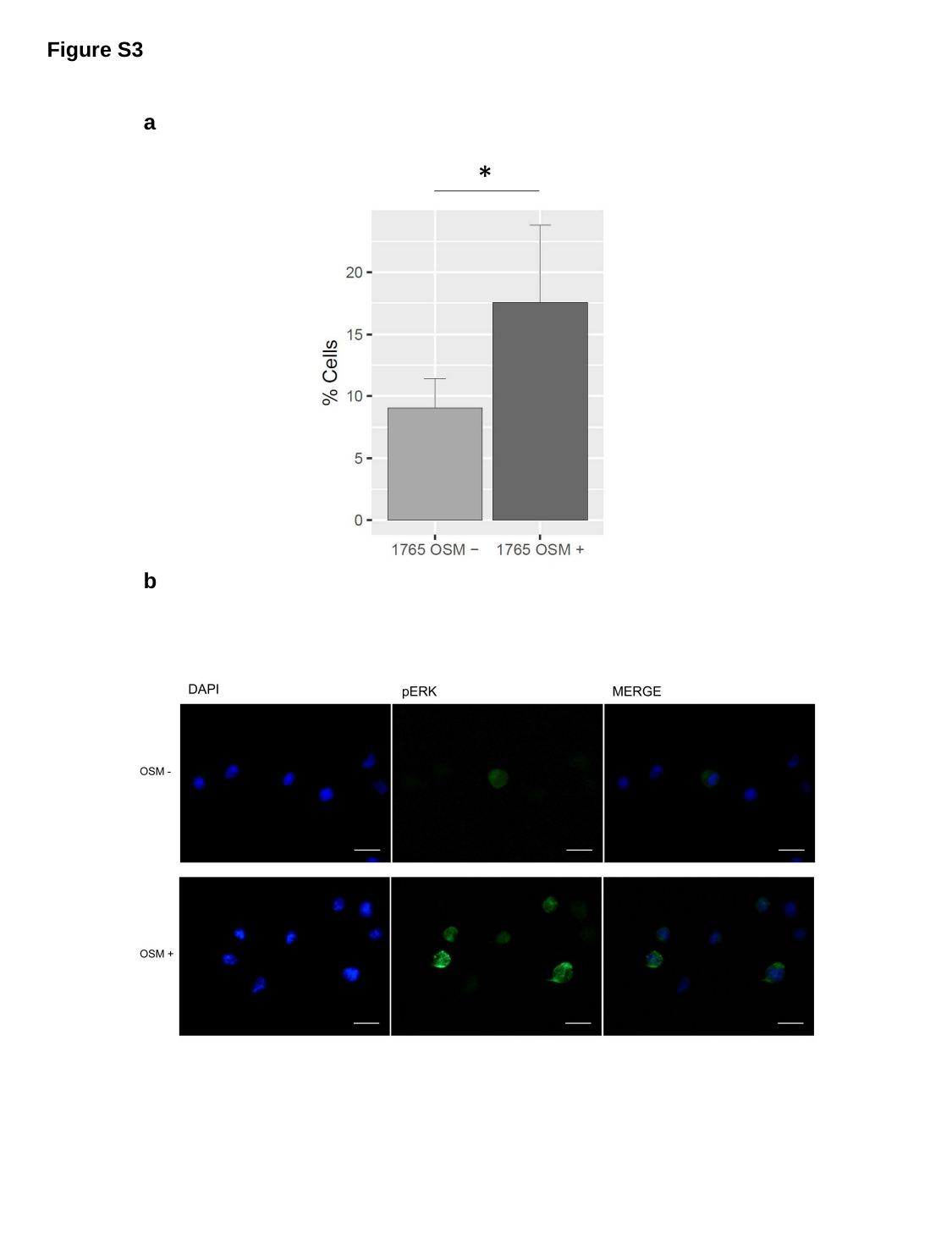

Figure S3
a
b

Supplement: Supplementary file 1 [file ijms-22-03831-s001.zip › FigureS3_IJMS_Lantieri.pptx]

## Slide 1
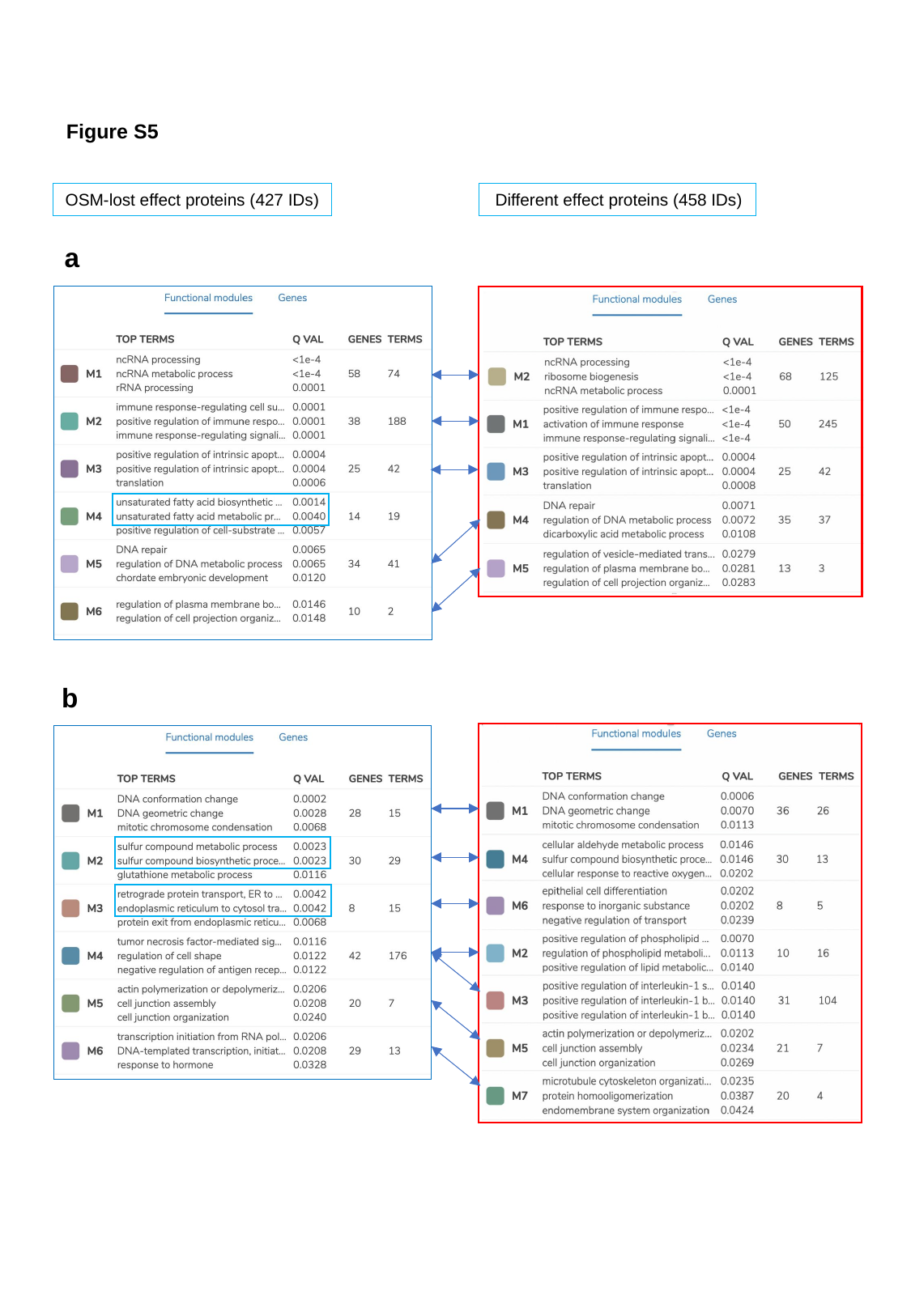

Figure S5
OSM-lost effect proteins (427 IDs)
 Different effect proteins (458 IDs)
a
b

Supplement: Supplementary file 1 [file ijms-22-03831-s001.zip › FigureS5_IJMS_Lantieri.pptx]

## Slide 1
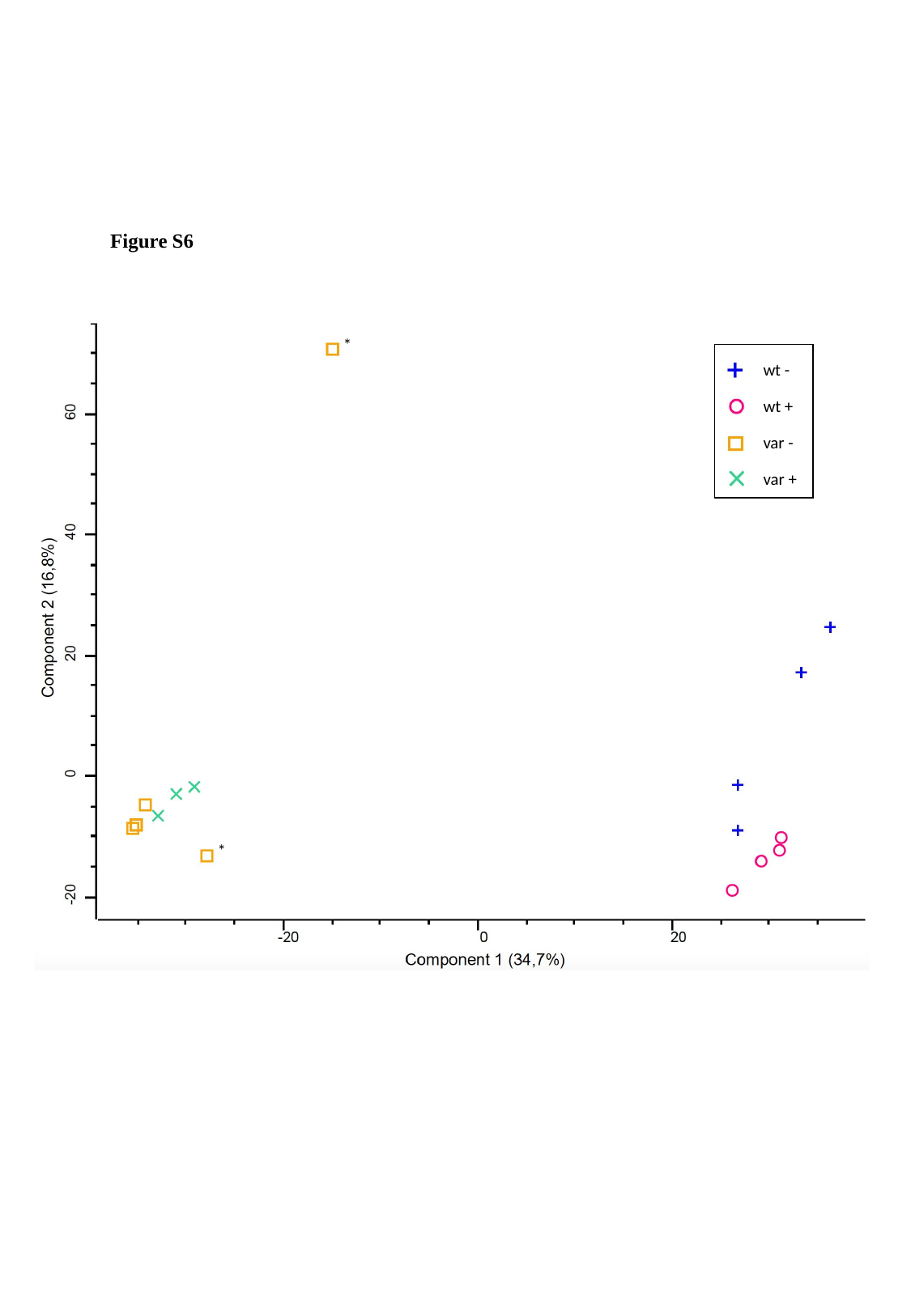

Figure S6
*
wt -
wt +
var -
var +
*

Supplement: Supplementary file 1 [file ijms-22-03831-s001.zip › FigureS6_IJMS_Lantieri.pptx]

## Slide 1
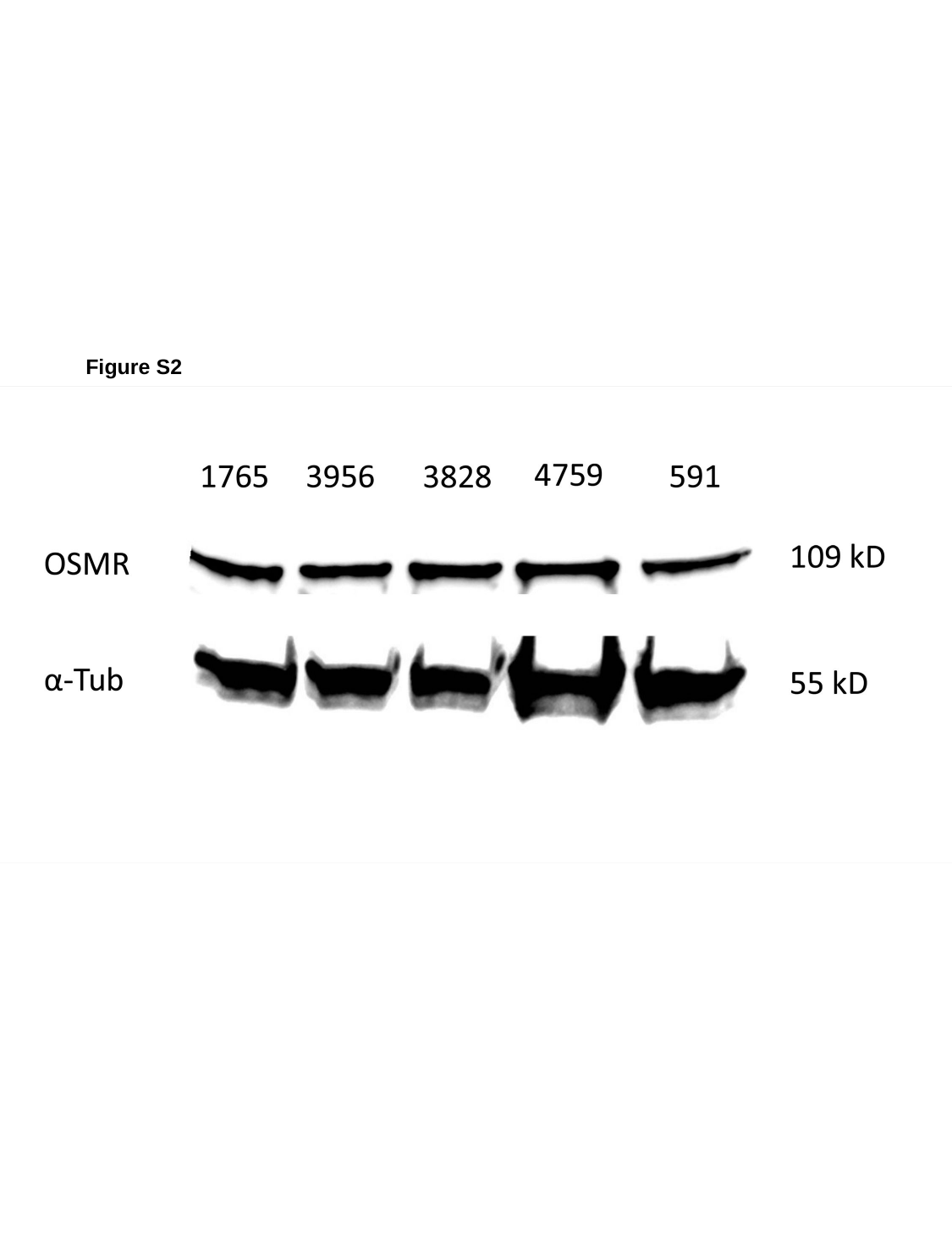

Figure S2

Supplement: Supplementary file 1 [file ijms-22-03831-s001.zip › FigureS2_IJMS_Lantieri.pptx]

## Slide 1
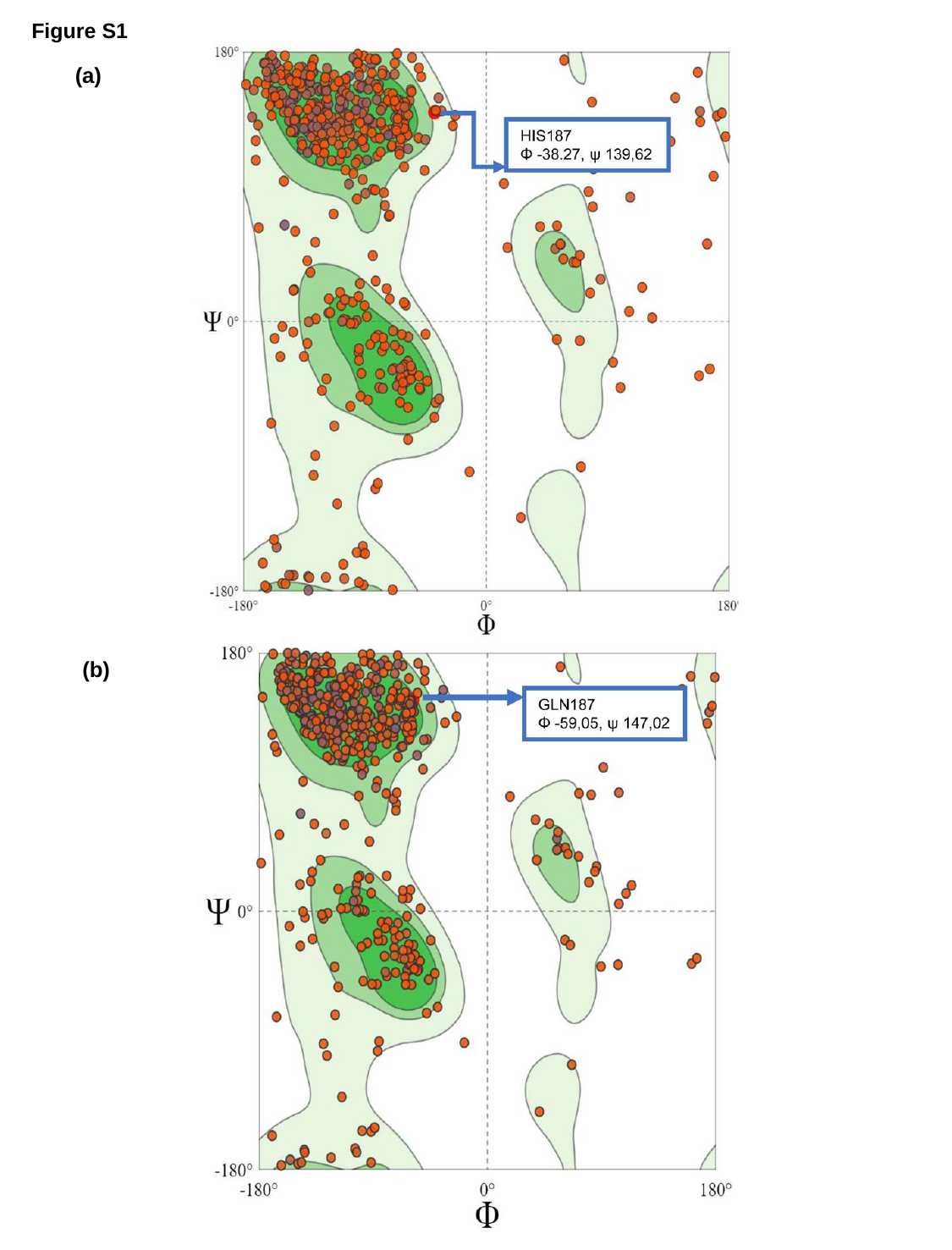

Figure S1
(a)
(b)

Supplement: Supplementary file 1 [file ijms-22-03831-s001.zip › FigureS1_IJMS_Lantieri.pptx]
